# Palpitation; Its Diagnostic Value

**Published:** 1871-01

**Authors:** J. Milner Fothergill

**Affiliations:** Senior Resident Medical Officer of Leeds Public Dispensary; Leeds


*Palpitation: its Diagnostic Value.* By J. Milner Fothergill, M.D., Senior Resident Medical Officer of Leeds Public Dispensary.

Though the oldest known symptom of heart disorder, palpitation has never had any exact diagnostic value attached to it. It has been hitherto regarded as excited or increased action of the heart, without any reference to its causation. It may be fairly questioned whether this even is in accordance with fact. From a careful consideration of the subject for some time past, I have been led to regard it as solely diagnostic of a disturbance of the balance which exists between the blood to be driven by the heart and the power to drive it; and that, too, in the direction of cardiac inability. This impression is constantly growing stronger. The idea is founded not only on its exciting causes, but also on the known action of the remedies which enjoy the greatest reputation for efficacy in allaying it. It appears to be the first evidence of over-taxation of the heart, of which irregularity (in time, not in volume—that rather belongs to mitral regurgitation) is the next, and intermittency the most serious.* This idea may appear more or less novel, but probably less. Having been for some time past engaged in an investigation into the mode and manner of production and action of heart disorder and disease, it may not be presumptuous to offer my conclusions, and why I have formed them, to the notice of the profession.

Palpitation, then, to commence broadly, is usually associated with exertion, nervous excitement, or general exhaustion, no matter how produced. It can be no subject for doubt that many persons know palpitation only after excitement in a heart structurally sound, others after exertion in a heart structurally diseased. But in each it may be the chief burden of complaint; indeed, in the person with the sound heart it may be more severe and alarming. Though connected with such apparently different conditions, its diagnostic value is unaltered. It has been regarded as over-action: the truth seems to be, rather, that action is more apparent. The extreme of healthy action is utter unconsciousness that such action exists. The evidence of that action, then, is scarcely to be regarded as an evidence of power. Though hypertrophy and palpitation are often found together, palpitation is much more inti-

* It is the intention of the writer to issue other papers on the subject of Irregularity (i e., loss of rhythm) and Intermittency (i. e., loss of one or several beats from either absence of ventricular contraction or a contraction so feeble as to be impracticable), from time to time, as leisure will permit.

mately associated with dilatation. Hypertrophy evidences that there is some disturbing force in operation by which increased action and power on the part of the heart are called for; otherwise hypertrophy could not exist. We are unacquainted with any growth of muscular fibre which is not occasioned by increase of demand upon it. In the silently acting organic muscular fibre this is most apparent. Thus it occurs in the stomach before a pylorus narrowed by scirrhus; in the bowel in the presence of a chronic obstruction; in the bladder from prostatic change or stricture. So in the heart we have hypertrophy from the demand for it, and can no more conceive a spontaneous, uncalled-for hypertrophy of muscular fibre in the heart than anywhere else. Palpitation evidences the demand, and indicates the compensation to be still incomplete. But it is connected with the demand on the heart; not called forth by the increase of muscular power. Of old, hypertrophy was regarded as a disease, and was met by bleeding and starvation. It was regarded as an abnormal development of power, and, as such, was attacked as a disease. We now know that it is a compensatory growth; and, consequently, the more perfect the hypertrophy the less does the patient or any one else hear or know of it. So palpitation, which has been regarded as over-action, and treated accordingly, will soon be seen to be an evidence of the muscular power being taxed or wanting, and that it is connected with the want of power, not the excess of it. Palpitation is, like the pain in the distended bladder, an evidence that the muscular action is called on; but it is not that it is too strong for the work, but that it is unequal to it. In dilatation, with or without valvular disease, is palpitation most common in the structurally altered heart. It cannot, then, be an evidence of over-action, as the old theory ran; because then the palpitation should be absent on exertion and present in quietude. Now we all know that such is not the case. In this condition effort calls out palpitation, as occasioning an addition to the heart's labor. The muscles cross the arteries and impede the flow of the blood. Wardrop even called this the musculo-cardiac function, and thought it beneficial by filling the heart's chambers with blood, which it most unquestionably does. But in dilatation the essence of the disease is that the ventricles are always too full, and never completely emptied. The ventricle, no longer able to close completely in systole, is partially full to commence with on diastole, the distended auricle and veins drive in the blood under increased pressure, the fibres become over-stretched, distension results, and contraction is impaired. "Dilatation of the heart is a purely mechanical effect of over-distension." Palpitation, then, ought, on the old theory, to act as a curative agent in dilatation, and ought consistently to occur when there is no great call on the heart—that is, during quietude, when the demand is the least.

But notoriously it occurs in exertion, when the impeded flow of blood would act against this over-action. This creed is untenable, and may be left without further comment to the decision of my readers. In the structurally diseased heart effort is the common cause of palpitation. A species of balance has been struck between the power of the heart and the work to be performed; the system has lowered to meet the heart; a leveling-down process has gone on; and effort, by disturbing this balance, evokes palpitation. Palpitation evidences that the muscular fibres are making an effort to meet the demand on them; not that they are inordinately successful. It is laboriousness, not excessive power, that is indicated by palpitation. When the heart is acting excitedly, as in exertion, it is only when that exertion has been excessive that anything like palpitation is evoked. Take a "striker" (a fore-hammerman), for instance, after a "heat." All his muscular energy is called out to accomplish all that it is possible to do before the metal cools. Take him at the finish, with the perspiration rolling down, panting, exhausted for the moment, and you find his heart palpitating, so violent is its action from the recent strain upon it. But its effect is visible on the pulse, which is full and bounding, and in strict proportion to the action of the heart; and there it differs from palpitation. In excitement a similar violent action is seen sometimes, but the arterial pulse is again affected and reveals the excitement. But these conditions are not true palpitation. According to the old theory, palpitation ought to occur to the striker when unemployed, as the heart then could not have enough to do. Of course some readers may say, "And so it does." Certainly in time it does, as it does in any one else where violent calls on the heart are inducing structural change According to the old theory, the heart, accustomed to exertion, ought to palpitate whenever a lessened call on it left it with some spare power, which is let off harmlessly as palpitation. So also in exertion, the heart's action ought to be feebler in proportion to the obstruction offered. Whether what is consistent with the old theory is consistent with experience may be fairly questioned.

But, again, it may be said that palpitation occurs from excitement, and is nervous. But when we have eliminated the cases of Bright's disease and those so-called hysteric conditions accompanied by a contraction in the arteries and arterioles, the instances will be found to be very few, and to depend mainly on the presence of poisons, either taking their origin within the organism, as in bile-poisoning, for instance, or from the action of external agents, as nicotine. It is possible that there may exist conditions essentially nervous, where a derangement of the balance of nerve-supply to the heart, a disturbance of the balance existing between the cardiac ganglia of the sympathetic and the inhibitory action of the pneumogastric, may occasion palpitation; but of these conditions we do not yet know anything clinically. Nervous palpitation is a refuge in time of trouble only too commonly. Increasing knowledge of pathological conditions, and the advance of our acquaintance with the mode of action of disordered function, are shedding much light on what have been hitherto obscure cardiac derangements. Firstly came the connection between disease of the kidneys and heart-changes. Traube attributed them to obstruction to the flow through the renal artery acting on the general systemic circulation; then came the idea of altered blood—in plain English, increased friction; and now Dr. Geo. Johnson has demonstrated a thickened muscular coat in the arterioles. The last theory supposes that the irritant action of the products of histolysis in the blood gives rise to a spasmodic action of the muscular tunic, in time leading inevitably, by the well-known law, to hypertrophy. Thus still greater obstruction is offered to the flow of blood, and the heart still more taxed. Thus when the blood is more than ordinarily laden with the products of retrograde tissue-metamorphosis, the excess irritates the arterioles, and more than usual contraction follows, and, as a consequenee, impeded flow and palpitation. The balance existing between the altered muscular tunics and the heart's walls, also altered, is disturbed and the accommodation interfered with, and the extra call for action evokes palpitation. As to the frequency of palpitation among those suffering from Bright's disease, and its occurrence along with evidence from other sources of an accumulation of retrograding material in the blood, as a question of fact, I must leave to the experience of my readers.

In hysteria and allied nervous disorders we have palpitation without evidence of any structural change. So also it occurs in persons of bilious temperaments; it follows excessive tobacco-smoking in nervous or weak persons, as it may also follow the administration of other agents which affect the heart. Whenever there is disturbance in the balance of nerve-forces it may occur. Whenever the heart is acting under disadvantageous circumstances it is never long absent. Thus palpitation follows displacement, either transient or lasting. It persists in displacement from accident or disease. It is a common concomitant of a loaded stomach or great flatulence. Indeed, it may arise from any cause which, by pressure on the diaphragm, diminishes the space for the heart and impedes its beat. This looks rather like a laborious action than over-action or spare power. The heart is placed at a disadvantage, and palpitation takes the place of normal quiet contraction.

Palpitation is intimately associated with a disturbed or altered innervation; whenever, in fact, there is a disturbance in the balance of the heart's nerve forces it may occur. In many cases of nervous palpitation—it would, perhaps, be thought premature to say all—no increased action is felt in the arteries, great or small. Whatever nerve-derangement may induce the palpitation, there is no corresponding action in the arteries. The condition may be analogous to, and even homologous with, the disordered action of Graves' disease. In Graves' disease, and in some cases of chorea, the whole right ventricle seems violently thrown against the chest-walls with a large and forcible impulse; the impulse, too, being in the normal locality, not the large diffuse beat of dilatation a rib or so lower than natural. Certain it is that, not uncommonly, a violent action of the heart exists where the pulse is small, and even unusually constricted. This condition is common in women. It is evident, then, that, in this condition, where even the sphygmograph may not detect an unusual action in the arteries, the heart's action must be met or neutralized by some altered condition of the arteries. In some cases, too, there is no excitement in the large arteries, showing that the arterioles are not the only part affected. In hysteria we usually see this condition: the heart is acting with evident violence against no perceptible necessity. The patient is cold, the pulse small, the arteries evidently contracted, and the state of arterial tension is followed by a free flow of limpid urine, the result of an increased pressure on the glomeruli of the kidney. This condition has met with much elucidation from experiments performed by Ludwig, Thierry, the brothers Cyon, and von Bezold. (See "Carpenter's Human Physiology," 7th ed., p. 269.) In these experiments an excited action of the heart was evoked after every nerve-communication had been severed between the heart and the organism, the heart only being attached by its vessels. Irritation then applied to the medulla oblongata induced violent action of the heart, increasing its pulsations in rapidity. When only a small portion of the nerve-supply, the accelerator nerve, was left, there was increase of power as well as of rapidity—i. e., typical palpitation.

These experiments have thrown much light on the pathogeny of nervous palpitation, and from them we can easily see how and when long continued tissue-changes will result. Thus is produced the nervous hypertrophy of writers. When, then, we find the arteries unaffected by the heart's action, it is evident that that action is met or neutralized by a condition existing in the arteries. That this condition is connected with vaso-motor changes in the calibre of the arteries and arterioles we are fast learning to see. Thus, in nervous hypertrophy we see the effects of opposition offered to the flow of blood continued for some time. In nervous palpitation we see evidence of an extra demand on the heart, and of action to meet it. In nervous palpitation we are no longer driven to imagine a tumultuous action of the heart's fibres against each other—a condition which it is not easy to imagine, much less to offer any proof of. In the uterus sometimes is seen a want of synchronicity between the action of the different fibres, which was supposed to resemble palpitation; but even then it was merely a spasmodic action of a few fibres, not a tumultuous action of them all. If there is this antagonistic action in the fibres of the heart, it is certainly, so far as I know, unique, and foreign to the action of all other collections of fibres. In fact, there appears an inherent improbability in the possibility of its existence even; and such an explanation can only be admitted in default of any other one, and when every other known cause has been looked for and found wanting. Such an association is not associated with scrutiny, but with the want of it. I believe we will find that we can no more have a spontaneous, uncalled-for palpitation than we can have an uncalled-for, spontaneous hypertrophy, though not long ago that was supposed to be a common occurrence. As hypertrophy has been found to be a compensatory change, so will palpitation be found to be merely a symptom of over-taxation, and that only.

As palpitation occurs in structurally altered hearts on exertion, so it will be found, in nervous affections (so called) of the heart, to be the evidence of the heart's struggling against some opposition offered to the flow of blood. It is scarcely credible that palpitation can be connected with what is so obviously effort in the one case, and be simply an evidence of excess of power in another case. That palpitation may be occasioned by external agents, as nicotine or white hellebore, which French soldiers used to take to stimulate heart disease, or any other agent which will disturb the nerve-balance, is as unquestionable as that it may also be relieved by other external agents. Thus, though palpitation may be allayed, and a partial palsy produced, by morphia or prussic acid, the remedies which are in most repute are diffusible stimulants, digitalis, and belladonna. Experiments on the frog have convinced me of what my clinical experience had long taught—namely, that digitalis and belladonna are really cardiac tonics—that is, they aid the ventricular contraction. They thus enable the heart to act more powerfully against the resistance offered, and in evercoming it the heart becomes quiet, regular, and normal; the quiet action or ability to act has taken the place of tumultuous effort; the ventricle can now contract without the visible effort—palpitation. That the effect of these agents is not to paralyze the heart's efforts is evident from the increased strength which is obtained by their exhibition. The relief is not due to their palsying effort, but to their aiding contraction.

Palpitation may be due to a call on the right ventricle, where the excessive action (if it were so) of the ventricle would not cause any effect in the radial pulse, or systematic arteries. But to meet the question of palpitation by attributing it to an activity of the right ventricle only, in cases where effect on the arteries may be wanting, is simply to shirk the broad issue. We know that the symptoms of over-action and demand on the right ventricle are widely different from those of simple palpitation; and that a totally opposite series of symptoms are then presented. We can scarcely, then, attribute palpitation to an over-action of the right ventricle only. That we may have a strong action of the right ventricle evoked when there is no stress upon the left ventricle, is true; but in that case again it is effort to overcome obstruction. The fibres of the heart are, in many instances, common to both ventricles, and excited action in one cannot exist without an approximating condition of the other ventricle.

In considering the question of nervous palpitation without corresponding effect upon the arteries, we must remember that the cardiac ganglia act against the inhibitory action of the vagus, as well as against the blood-column. It is possible, then, that there may be a disturbance of the balance between them. But then there would be a long irregular systole, as is seen when the balance is disturbed by poisons. In this condition the muscular fibres can take no part, except in acting in accordance with the nerve-force brought to bear on them. But in this disturbance of the nerve-balance the action is irregular, slow, and intermitting, according as some ganglia are more or less acted upon, and bears no resemblance to palpitation. Neither does galvanizing the vagus, as done by Weber, act in a manner at all analogous to palpitation. There is arrest of contraction, with one or two powerful beats on cessation of the stimulus; but this is not palpitation.

In palpitation from dreaded loss of blood, as in uterine hæmorrhage, we have what may truly be called over-action, by a reduction in the opposition offered to the ventricular contraction; the ventricular action is not excessive, but the blood no longer offers its wonted opposition. Now, muscular action must produce results either in overcoming opposition offered, or in natural contraction of itself—i. e., spasm. When, then, in this condition the muscular fibres are less distended by influx of blood, the energy is manifested in abnormal contraction, and ends finally in confirmed systole, as occurs in animals bled to death. The heart is here firmly contracted until it is impossible to contract further, but during this time the animal has died. Muscular action cannot go on without result; and if we have an action in the muscular fibres which is not met by some opposing force, unnatural contraction, or spasm, must ensue; and if palpitation were truly over-action of the heart, it would be one of the most dangerous and fatal of conditions. The further the inquiry is pushed, the less evidence can we find in favor of palpitation being over-action of the heart: it is a laborious effort, either from increased demand on the heart, or unfitness in itself. The heart, when its integrity is impaired or it is exhausted by effort, palpitates—i. e., a laborious evident effort takes the place of the steady normal condition. If the obstruction offered is not at first sight apparent, it is nevertheless there. Muscular action cannot be barren in results: the ventricular action must either be apparent in the arteries, be neutralized by some condition existing in them, or end in self-contraction.

Palpitation, as a temporary evidence of over-taxation, may be engrafted on chronic conditions of acknowledged failure of power. Thus, in valvular disease, or dilatation, we may have irregularity, or even intermittency, as persistent evidences of cardiac asthenia, and along with this an enfeebled general condition: that a balance has been struck by the lowering of the general condition to meet the heart's enfeebled state. On this we may have implanted palpitation on exertion, as evidencing a temporary overthrow of that balance; no pathological condition is more common in fact. If, then, palpitation were truly over-action of the heart, the sufferer ought to feel more equal to exertion during its occurrence; it ought to restore again to him that power the want of which has so crippled him. In asthenic conditions palpitation ought to be a tonic, and it ought also to occur most frequently among the robust and those accustomed to exertion;—i. e., palpitation ought to occur when the heart has not its accustomed exertion to undergo, during general quietude. Whether such is the case, or palpitation is found to occur on or after exertion in asthenic conditions, I will leave to others to decide, as it is a question of fact. My own experience has been unequivocal. As not being normal action, it must be connected with either excess or deficiency of power. As to whether it is associated I leave to our common experience. If it be allowable to anticipate and pry into the future, it may safely be predicted that in time we shall come to learn that palpitation is as surely the evidence of over-taxation of the heart's powers, as we have learnt that cardiac hypertrophy is a compensatory growth, the result of a persistent call upon the heart for augmented effort. "It must be recollected that, in every organic disease of the heart, when palpitation becomes extremely violent and prolonged, both the impulse and the sounds may be diminished: in other words, the heart becomes gorged and incapable of adequately contracting on its contents, sometimes yielding a struggling convulsive impulse, with little sound and a feeble pulse, and, in an ulterior degree, especially during dissolution, scarcely producing either impulse, sound, or pulse. Suffocative dyspnœa, lividity, and extreme distress, are always concomitant symptoms." So wrote Hope, even twenty years ago.—*London Lancet.*

LEEDS, July, 1870.

